# Identification of volatile lung cancer markers by gas chromatography–mass spectrometry: comparison with discrimination by canines

**DOI:** 10.1007/s00216-012-6102-8

**Published:** 2012-06-03

**Authors:** Bogusław Buszewski, Tomasz Ligor, Tadeusz Jezierski, Anna Wenda-Piesik, Marta Walczak, Joanna Rudnicka

**Affiliations:** 1 Chair of Environmental Chemistry and Bioanalysis, Faculty of Chemistry, Nicolaus Copernicus University, 7 Gagarin St, 87-100 Toruń, Poland; 2 Department of Animal Behaviour, Institute of Genetics and Animal Breeding of Polish Academy of Sciences, Jastrzebiec, 05-552 Wólka Kosowska, Poland; 3 Department of Plant Growth Principles and Experimental Methodology, University of Technology and Life Sciences, 20 Kordeckiego St, 85-225 Bydgoszcz, Poland

**Keywords:** Lung cancer, Canine olfaction, GC–MS

## Abstract

In this work, a chromatographic method for identification of volatile organic compounds was compared with canine recognition. Gas chromatography and mass spectrometry (GC–TOF MS) were used for determination of concentrations of trace gases present in human breath. The technique enables rapid determination of compounds in human breath, at the parts per billion level. Linear correlations were from 0.83–234.05 ppb, the limit of detection was the range 0.31–0.75 ppb, and precision, expressed as relative standard deviation (RSD), was less than 10.00 %. Moreover, trained dogs are able to discriminate breath samples of patients with diagnosed cancer. We found a positive correlation between dog indications and the ethyl acetate and 2-pentanone content of breath (*r* = 0.85 and *r* = 0.97, respectively). The methods presented for detection of lung cancer markers in exhaled air could be used as a potential non-invasive tool for screening. In addition, the canine method is relatively simple and inexpensive in comparison with chromatography.

## Introduction

Lung cancer is one of the most common malicious tumours and one of the main causes of death in developed countries. The predominant factor that contributes to lung cancer is smoking, both active and passive, because cigarette smoke contains over the 200 substances with carcinogenic or mutagenic activity. Exposure to radon, cadmium, arsenic, beryllium, or asbestos is another common cause of the disease.

Lung cancer is classified into two broad groups: small-cell lung carcinoma (SCLC) (20–25 % frequency of occurrence) and non-small-cell lung carcinoma (NSCLC) (70–75 %). The latter category includes adenocarcinomas (25–30 %), squamous cell (30–35 %), and large-cell carcinomas (10–15 %). The classification takes into account the histological type of the cancer, the method of treatment, and tumour prognosis [1–4]. High lung cancer mortality is primarily because of late diagnosis. Regular screening for early lung cancer symptoms is a promising way of reducing mortality. Therefore, finding non-invasive, painless, and easily accessible screening techniques facilitating early diagnosis are important objectives.

Determination of volatile organic compounds (VOCs) in exhaled breath can provide valuable information about the condition of human health. Numerous analytical techniques, for example sensor arrays proton transfer reaction mass spectrometry (PTR MS), selected ion flow tube mass spectrometry (SIFT MS), and tunable diode laser absorption spectroscopy (TLDS), have been used for this approach [5–14]. However, chromatography mass spectrometry (GC–MS) is still the most useful [15, 16].

Williams and Church reported cases in which untrained pet dogs warned their owners about thigh lesions. The lesions were later diagnosed as malignant melanoma [17, 18]. These authors were the first to propose the idea that dogs were able to detect human cancer on the basis of odour. The dogs spontaneously demonstrated persistent interest in their owner’s leg by sniffing, licking, and trying to bite the lesions off even through clothing. When the carcinoma lesions were excised, the dogs showed no further interest in the site. Other papers report the ability of specially trained dogs to distinguish, on the basis of odour, samples taken from patients suffering from cancer from those taken from healthy humans [19]. Thus, dogs could be used for the detection of different kinds of neoplastic disease, e.g. melanoma, lung, breast, prostate, or ovarian cancer [20–23]. Balseiro and Correia hypothesized that the volatile organic compounds produced by tumours and detected by dogs are the products of the major histocompatibility complex (MHC) genes [24].

Use of sniffer dogs has some advantages compared with contemporary analytical methods for identification of VOCs, for example chromatography or mass spectrometry (GC–MS). Furthermore, dogs’ mobility enables detection in different sites outside a laboratory. A trained dog’s response to a detected odour (sitting or lying down) is binary in character, i.e. in the form of clear-cut yes/no response, which makes interpretation of the results much simpler.

Use of gas chromatography and mass spectrometry (GC–MS) for cancer screening may, in practice, be problematic, not only because of the sampling procedure but also because of difficult interpretation of the results.

A variable number of VOCs may be identified in the breath of patients with diagnosed cancer. Some compounds may be present in different combinations and quantities. Therefore, use of a single VOC as a cancer marker seems useless [25–28]. Because several markers in combination could enable better diagnosis of the disease, sophisticated methods of multivariate analysis, for example fuzzy logic have been applied to the results obtained from GC–MS. On the other hand, black-box technology is a serious drawback of canine indications in a scent line-up; it is unknown which single odour or combination of odours dogs respond to.

In this paper, experiments which involved using trained dogs to detect odour markers of lung cancer in breath samples are reported. The results obtained by recognition by dogs are also compared with those obtained by GC–MS.

## Experimental

### Materials and reagents

Analysis was performed with an Agilent (Waldbronn, Germany) 7890A gas chromatograph coupled with a TruTOF spectrometer (Leco, St Joseph, USA). The system was equipped with a CP-Porabond-Q (Agilent) 25 m × 0.25 m × 3 μm column. The oven temperature programme was: initial temperature 40 °C for 2 min, then ramped at 10 ° min^−1^ to 140 °C, then ramped at 5 ° min^−1^ to 270 °C which was maintained for 5 min. The temperature of the split–splitless injector was 200 °C. Electron impact (EI) spectra were acquired in the mass range 30–300 *m*/*z*; the electron energy was 70 eV and the acquisition rate was 50 spectra s^−1^. Both ion source and transfer-line temperatures were 200 °C. Collection of chromatographic data was performed by means of ChromaTOF software (Leco). A carboxen–polydimethylsiloxane-coated fibre (Supelco, Steinheim, Germany) were used for SPME.

All chromatographic standards (aldehydes, alcohols, hydrocarbons, and ketones) were purchased from Sigma–Aldrich (Steinheim, Germany).

### Breath collection

Breath samples were collected from 44 healthy volunteers and 29 patients with lung cancer (including 18 people with SCLC and 11 with NSCLC, all volunteers). Breath samples were collected in the Collegium Medicum, NCU. The study was approved by the local ethics commission. In each patient’s case, a questionnaire on cancer and its stage was completed. Data such as age, sex, other diseases, prescribed drugs, smoking habits, and information about previously consumed meal were also collected.

The alveolar breath samples were collected by means of a CO_2_ controlled sampler (Department of Anesthesiology and General Intensive Care, Innsbruck Medical University, Innsbruck, Austria) in 1-L Tedlar bags. Before collection of breath, all bags were cleaned by flushing with argon gas and then filled with argon and heated at 60 °C for 12 h to remove any contamination. A 200-mL sample was then transferred into a second bag. The SPME fibre was introduced into the bag through a septum and exposed to a sample. After extraction for 15 min the SPME fibre was desorbed in the hot GC injection port for 2 min at 200 °C. Ambient air samples were taken for blank measurement.

### Standard preparation

A gaseous standard was prepared by injecting 1 or 3 μL of liquid compounds into a 1-L glass bulb. The liquid was then evaporated. The mixture was subsequently diluted in Tedlar bags filled with nitrogen to obtain concentration in the range 0.3–234 ppb.

### Experiments with sniffer dogs

Male dogs (German shepherd mix) that successfully underwent three-phase training in the scent line-up were used for lung cancer detection. The dogs were 20-22 months old.

The experimental procedure and the conditions in which the dogs were kept were approved by the Local Ethics Commission for Animal Experimentation in Warsaw. For testing with the use of dogs, breath odour samples were collected from the same donors and at the same time as for the GC–MS analysis. The odour samples for canine training and detection were taken by exhaling 2 or 3 times through disposable polypropylene sampling tubes (Defencetek, Pretoria, South Africa) 15 cm long and 3 cm in diameter.

### Sniffing testing procedure

To test the samples, the removable inserts were taken out of the tubes and placed in sterile polypropylene boxes covered with hole-punched lids to prevent direct contact of a dog’s nose with the insertion, salivation, etc.

One breath odour sample taken from a patient with lung cancer was placed in a line-up with four samples from healthy volunteers (controls). The dogs were taught to indicate the lung cancer sample by the sitting-down response in front of the sample. The trials with dogs were repeated approximately 30 times on different days. The positions of odour samples in the line-up were randomly changed for every trial. To prevent suggestion of answers to the dogs during detection, the experimenter was invisible to the dog, and the dog handler was not aware of the cancer sample position in the line-up.

## Results and discussion

The linearity, precision, and detection limits for determination of VOCs in human breath are shown in Table 1. The relative standard deviation (RSD) was in the range 3.3 to 9.5 % for hydrocarbons, alcohols, aldehydes, and ketones.

**Table 1:** Validation data for volatile organic compounds

Compound	Linearity (ppb)	*R* ^2^	RSD (%)	LOD (ppb)	LOQ (ppb)
Acetone	1.6–166.4	0.991	8.9	0.54	1.62
Acetonitrile	2.3–234.0	0.996	3.4	0.75	2.25
Benzene	1.3–136.7	0.995	4.6	0.43	1.29
Butanal	1.3–135.6	0.997	8.4	0.44	1.32
2-Butanone	1.3–136.5	0.997	3.3	0.45	1.35
Ethyl acetate	0.9–87.8	0.992	4.5	0.32	0.96
Ethylbenzene	1.0–99.8	0.992	4.5	0.32	0.96
Furan	1.6–165.6	0.999	4.9	0.51	1.53
Hexane	1.5–150.0	0.994	3.4	0.48	1.44
2-Methylpentane	0.9–92.6	0.995	9.5	0.31	0.93
3-Methylpentane	0.9–94.1	0.988	9.3	0.32	0.94
Pentane	1.5–150.0	0.998	5.2	0.49	1.47
2-Pentanone	1.3–103.7	0.996	6.2	0.44	1.32
Propanal	1.7–170.4	0.998	7.2	0.52	1.56
1-Propanol	1.6–163.5	0.995	5.1	0.53	1.58
2-Propanol	1.6–159.6	0.998	9.4	0.52	1.57
2-Propenal	1.3–133.9	0.996	6.2	0.44	1.32
Toluene	1.1–114.7	0.991	5.9	0.37	1.11
*o*-Xylene	1.0–100.1	0.994	4.8	0.33	0.99

R^2^, correlation coefficient ; RSD, relative standard deviation ; LOD, limit of detection ; LOQ, limit of quantification

Calibration curves were linear for aliphatic hydrocarbons in the range 0.9–150.0 ppb, for alcohols 1.6–163.5 ppb, for aldehydes 1.3–170.4 ppb, for ketones 1.3–166.4 ppb. Regression coefficients were high, at least 0.991. The lowest LOD values, obtained for hydrocarbons, varied from 0.31 to 0.49 ppb.

### Exhaled air

**Fig. 1:**
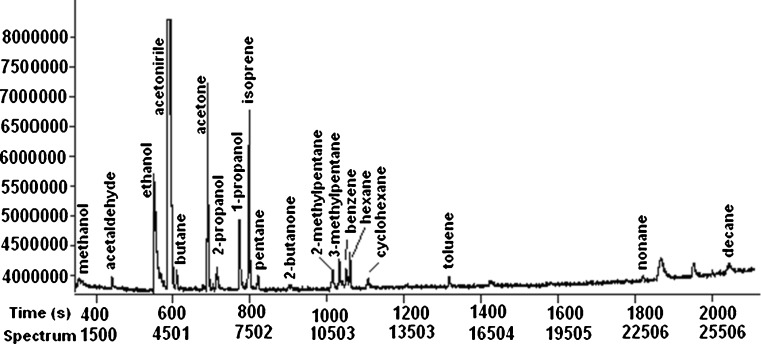
GC–TOF MS chromatogram obtained from a sample of air exhaled by a person with lung cancer

All the compounds detected in breath samples were compared with ambient air samples, and only those compounds with concentrations at least 10 % higher than those in ambient air were reported. Figure 1 shows a chromatogram obtained from the breath of a lung cancer patient.

The concentration of pentane, which is regarded as an oxidative stress marker, ranged from 6.8 to 14.3 ppb for healthy people and from 0.7 to 17.5 ppb for patients with cancer (Table 2). Furan and its derivatives are regarded as smoking status markers. 1-Propanol was observed in breath of patients with cancer at concentrations in the range 4.37–13.15. This compound might be postulated as a potential cancer marker in breath [27]. Moreover, 2-propanol was found in healthy and ill people’s breath samples. Also, its concentrations in exhaled and ambient air were similar.

**Table 2:** Compounds detected in breath of healthy volunteers and cancer patients

Compound	Concentration range for healthy persons (ppb)	Concentration range for lung cancer patients (ppb)
Acetone	44.20–531.45	34.57–390.60
Acetonitrile	5.99–28.98	10.96–23.60
Benzene	1.38–14.97	1.29–3.82
Butanal	1.35–1.87	1.32–2.55
2-Butanone	1.35–3.18	1.35–2.86
Ethyl acetate	1.12 – 8.22	3.98 -22.89
Ethylbenzene	2.22–18.38	1.45–3.16
Furan	1.67–3.25	1.53–2.81
Hexane	1.75–6.31	1.44–1.88
2-Methylpentane	2.37–10.80	0.93–3.77
3-Methylpentane	1.05–8.76	0.94–8.87
Pentane	6.84–14.36	1.73–17.50
2-Pentanone	1.80–4.11	3.25 – 8.77
Propanal	1.56–3.44	1.56–3.74
1-Propanol	–	4.37–13.15
2-Propanol	3.21–4.17	3.32–7.19
2-Propenal	5.10–9.57	6.84–94.36
Toluene	1.45–37.21	1.12–17.10
*o*-Xylene	2.06–7.44	1.99–7.64

ppb, parts per billion

The original data from VOCs measurement did not meet the normality assumption of parametric ANOVA and even log transformation did not managed to produce a normal distribution for some compounds. Therefore, the alternative non-parametric ANOVA (Kruskal–Wallis test) was used to verify the null hypothesis assuming that three studied groups (cancer patients, control smokers, and control of non-smokers) came from the same population. This measures the probability that a random observation from one group is the same as a random observation from another group. The value of the chi^2^ test confirms the significance of an observation obtained in the Kruskal–Wallis test. For 12 substances the hypothesis of uniform concentrations of VOCs in patients’ breath and in that of healthy controls could be rejected. For the compounds shown in Table 3 the concentrations were significantly lower in the healthy group than in people with cancer. For butanal, 2-butanone, ethyl acetate, ethyl benzene, 2-pentanone, 1-propanol, and 2-propanol the tendency of greater concentration in the breath of cancer patients than in controls was found to be significant at *P* < 0.001.

**Table 3:** Kruskal–Wallis ANOVA by ranks for three groups (cancer patients, non-smokers, and smokers) with multiple comparisons for VOCs detected in breath air

VOC	Kruskal–Wallis test				*χ* ^2^	*P*	Cancer patients versus control group:	
	Groups	N	H	*P*			Non-smokers	Smokers
Acetone	3	74	7.13	<0.05	10.04	<0.01	↑	ns
Benzene	3	54	7.80	<0.05	7.96	<0.05	↑	ns
Butanal	3	62	37.40	<0.001	50.00	<0.001	↑ ↑ ↑	↑ ↑ ↑
2-Butanone	3	53	18.43	<0.001	20.75	<0.001	↑ ↑ ↑	↑
Ethyl acetate	3	62	37.40	<0.001	50.00	<0.001	↑ ↑ ↑	↑ ↑ ↑
Ethylbenzene	3	73	19.53	<0.001	16.61	<0.001	↑ ↑ ↑	↑
Furan	3	29	5.45	=0.05	6.34	<0.05	↑	ns
2-Pentanone	3	73	38.89	<0.001	33.97	<0.001	↑ ↑ ↑	↑ ↑ ↑
Propanal	3	29	13.39	<0.01	10.31	<0.01	↑ ↑ ↑	ns
1-Propanol	3	62	36.52	<0.001	44.18	<0.001	↑ ↑ ↑	↑ ↑ ↑
2-Propanol	3	65	44.09	<0.001	57.65	<0.001	↑ ↑ ↑	↑ ↑ ↑
2-Propenal	3	30	7.15	<0.05	4.80	=0.09	↑	ns

↑, elevated concentration in cancer group compared with control group at *P* = 0.05; ↑↑↑ elevated at *P* = 0.001

ns, not significant according to two-tailed test for corresponding multiple comparisons treatments versus control

### Dog experiments

Detection sensitivity and specificity were calculated by use of a yes/no response criterion toward each sniffed sample in the line-up, i.e. at 50 % probability of getting the correct response by chance. To evaluate differences in dogs’ indications between cancer and control samples, and the results obtained from particular dogs, the chi^2^ test was used.

The dogs indicated correctly the pattern of breath samples from lung cancer patients with detection sensitivity and specificity of 82.2 % and 82.4 %, respectively. False positive indications toward healthy controls amounted to 17.8 % of trials. The differences between dogs’ indications of cancer samples vs. controls were highly significant (chi^2^ = 1056, *P* < 0.000). There were significant differences between dogs in detection sensitivity (chi^2^ = 25.17, d.f. = 1, *P* < 0.001) but no significant differences in detection specificity.

### Correlation between a dog’s indications and chemical analysis

The data concerning the dogs’ indications were analysed. We tried to find the correlation between the VOCs in exhaled air and the dogs’ indications. Two data sets, for control people (*N* = 49) and for patients (*N* = 29), contained the percentage of a dog’s indications and the chosen compounds. For the patients group, positive Pearson’s correlations between the dogs’ indications and the VOCs content of breath had a significant positive or negative tendency, e.g. ethyl acetate and 2-pentanone correlated positively with the dog’s positive indications (*r* = 0.85 and *r* = 0.97, respectively), whereas for acetonitrile, propanal, and 1-propanol, the contents were negatively correlated with the dog’s indications (*r* = −0.78, *r* = −0.87, and *r* = −0.98 respectively). Two multivariate methods, FA and PCA, were used for data calculation, separately for the control and patient groups, with 19 dimensions presenting percentage of the dog’s indications and 18 for VOCs.

The data for each dimension were standardised within the individuals and, to obtain a meaningful structure of the principal components, the number of factors was finally limited to two. Factor analysis with varimax rotation was conducted as the first method for the datasets. For the patient group, two factors were essential for the classification with Eigenvalues larger than unity. These two factors provided an explanation for over 80 % of the total variation. The first factor had a positive value of the loading coefficient for ethanol, isobutane, butane, isoprene, pentane, and benzene. Simultaneously, negative values were obtained for carbon disulfide, 2-butanone, and toluene. The second factor was associated with a dog’s indication and the compounds which were not classified by the first factor.

Analogously to a simple correlation, the signs of loading explain the role of compounds in the dog’s indications in the same way as the simple correlations. Hence, the first factor could be named as indifferent for the dog, whereas the second factor correlated with the dog’s indications. Principal-components analysis (PCA) was also used for the classification to show the relationships between VOCs and the dog’s indications toward two components.

**Fig. 2:**
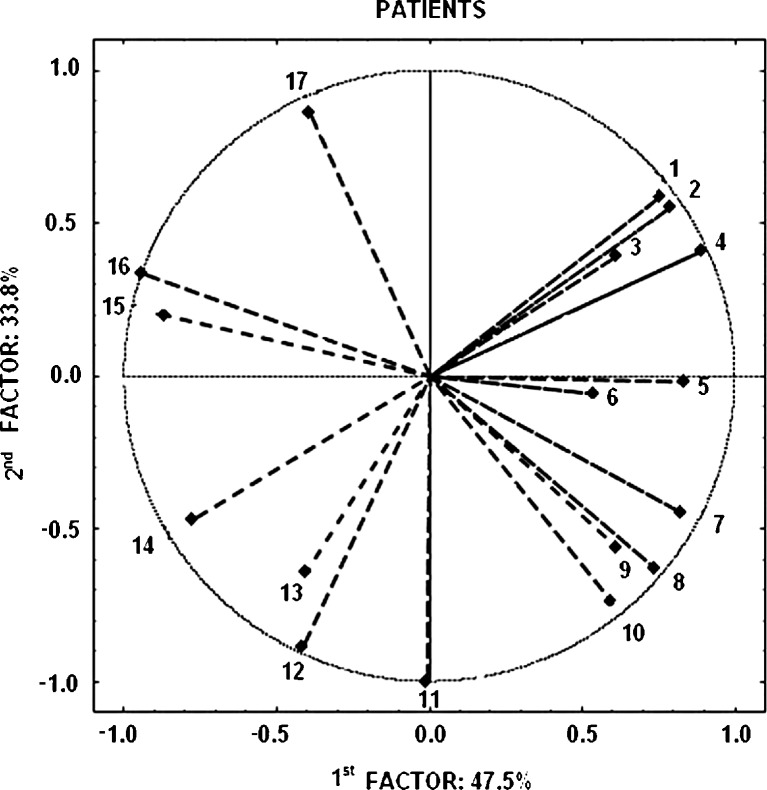
Compound classification based on dog’s indication: *1*, 3-methylpentane; *2*, ethyl acetate; *3*, hexane; *4*, 2-pentanone; *5*, pentane; *6*, 2-methylpentane; *7*, ethanol; *8*, butane; *9*, isobutane; *10*, benzene; *11*, 2-propanol; *12*, acetonitrile; *13*, propanal; *14*, 1-propanol; *15*, carbon disulfide; *16*, 2-butanone; *17*, toluene

Figure 2 enables the classification of the compound either to the first factor or to the second. For the control people two first factors were essential for classification but explained only 51 % of the variance. The interpretation of such results is uncertain.

It should be mentioned, however, that GC–MS analysis of particular VOCs does not explain how a mixture of compounds is perceived by canine olfaction. The odour signature of cancer that dogs use for discrimination of the samples may be related to some specific qualitative or quantitative olfactory impressions produced by a mixture of VOCs.

## Conclusions

Trained dogs are able to discriminate breath samples of patients with diagnosed cancer disease from those of healthy donors at a “better than by chance” rate. The canine method has the following advantages: dog training and testing is relatively simple and inexpensive in comparison with analytical equipment application (GC–MS) and detection sensitivity and specificity in relation to pattern samples were relatively high and easily interpretable. Although it is highly probable that dogs used specific breath odour to discriminate samples, it is still unknown which chemical compound(s) or odour mixture or other stimuli the dogs respond to. These investigations on parallel applications of GC–TOF MS and canine scent for the detection of volatile cancer markers show promise, however, because crucial biomarker candidates for non-invasive cancer screening might be found.
